# The Second-Generation XPO1 Inhibitor Eltanexor Inhibits Human Cytomegalovirus (HCMV) Replication and Promotes Type I Interferon Response

**DOI:** 10.3389/fmicb.2021.675112

**Published:** 2021-05-03

**Authors:** Yueyan Liao, Xiangyu Ke, Tianyi Deng, Qingsong Qin

**Affiliations:** ^1^Laboratory of Human Virology and Oncology, Shantou University Medical College, Shantou, China; ^2^Guangdong Provincial Key Laboratory of Infectious Diseases and Molecular Immunopathology, Shantou, China; ^3^Guangdong Provincial Key Laboratory for Diagnosis and Treatment of Breast Cancer, Shantou, China

**Keywords:** cytomegalovirus, type I interferon, replication, Eltanexor, XPO1 inhibitor

## Abstract

Human cytomegalovirus (HCMV) is a ubiquitous opportunistic pathogen and can be life-threatening for immunocompromised individuals. There is currently no available vaccine for the prevention of HCMV- associated diseases and most of the available antiviral drugs that target viral DNA synthesis become ineffective in treating HCMV mutants that arise after long-term use in immunocompromised patients. Here, we examined the effects of Eltanexor, a second-generation selective inhibitor of nuclear export (SINE), on HCMV replication. Eltanexor effectively inhibits HCMV replication in human foreskin fibroblasts in a dose-dependent manner. Eltanexor does not significantly inhibit viral entry and nuclear import of viral genomic DNA, but rather suppress the transcript and protein levels of viral immediate-early (IE), early (E) and late (L) genes, and abolishes the production of infectious virions. We further found Eltanexor treatment promotes proteasome-mediated degradation of XPO1, which contributes to the nuclear retention of interferon regulatory factor 3 (IRF-3), resulting in increased expression of type I interferon as well as interferon stimulating genes ISG15 and ISG54. This study reveals a novel antiviral mechanism of Eltanexor which suggests it has potential to inhibit a broad spectrum of viral pathogens.

## Introduction

Human cytomegalovirus (HCMV) is a β-herpesvirus, which infects up to 60–90% of the world’s population. HCMV infection is generally asymptomatic but can cause life-threatening complications in immunocompromised individuals, such as transplant recipients and patients with AIDS. It is also a leading cause of congenital infections (Cannon, 2019). Currently there is no vaccine for HCMV, and effects of antiviral therapy are limited due to the emergence of resistant viral mutants during a long-term treatment (Poole and James, 2018). Most antiviral agents are nucleoside analogs that target viral DNA synthesis. Unfortunately, antiviral therapy is hindered by the emergence of mutations in viral DNA polymerase UL54 and viral kinase UL97 which confer resistance to common anti-HCMV drugs such as ganciclovir and maribavir (Razonable, 2018). The newly FDA-approved anti-HCMV drug, letermovir, which targets the viral DNA terminase-complex, has been in use for a little over a year; however, resistant viral variants have already emerged (Douglas et al., 2020). Thus, there remains an urgent need to develop new antiviral therapies for HCMV.

Viruses are obligate intracellular agents and rely on cellular trafficking systems. Targeting the nuclear export machinery is a potential alternative strategy for antiviral therapies (Mathew and Ghildyal, 2017; Kosyna and Depping, 2018). Exportins are Ran-GTPase-dependent nuclear transport factors that belong to the karyopherin-β family and mediate the nuclear export of a plethora of proteins and RNAs (Matsuura, 2016). Exportin-1 (XPO1), also known as chromosome region maintenance 1 (CRM1), is a major receptor responsible for exporting proteins from the nucleus to the cytoplasm. XPO1 mediates nuclear export of more than 220 proteins including tumor suppressors and growth regulators such as p53, p21, FOXO, PI3K/AKT, Wnt/β-catenin, AP-1 and NF-κB, and is involved in the regulation of cell cycle progression and apoptosis (Fornerod et al., 1997; Xu et al., 2012). XPO1 binds to a diverse array of cargos in the presence of Ran-GTP via a nuclear export sequence (NES) composed of a cluster of leucine (L)-rich or hydrophobic amino acids (Dong et al., 2009; Monecke et al., 2009). Leptomycin B (LMB) is a prototypical inhibitor of XPO1 and covalently binds to Cys528 located in the NES binding groove of XPO1 (Kudo et al., 1999; Petosa et al., 2004; Monecke et al., 2009; Sun et al., 2013; Turner et al., 2014), and has been studied as a potent agent against various types of cancer as well as an antiviral agent for many years (Mathew and Ghildyal, 2017). However, LMB is unsuitable for therapeutic development, because it irreversibly shuts down nuclear export resulting in high cytotoxicity *in vivo* (Sun et al., 2013; London et al., 2014). This has led to the development of synthetic analogs of LMB (known as the second-generation selective inhibitors of nuclear export [SINEs]), such as KPT8602 (Eltanexor), KPT330 (Selinexor), KPT335 (Verdinexor), KPT185, that have substantially improved *in vivo* tolerance and are reversible (Ranganathan et al., 2012; Azmi et al., 2013; Gutierrez et al., 2013; Zhang et al., 2013; Zheng et al., 2014), and have been extensively tested in phase I/II clinical trials for solid tumors, and hematologic malignancies (Cornell et al., 2016; Hing et al., 2016).

SINEs have also been extensively studied for antiviral therapies as many viruses exploit or modulate XPO1-mediated nuclear export at various stages of their lifecycles (Gruffaz et al., 2019). It has been reported that SINEs inhibit the replication of numerous viruses including influenza virus (Perwitasari et al., 2014), HIV (Boons et al., 2015), Epstein-Barr virus, human cytomegalovirus, Kaposi’s sarcoma virus, adenoviruses, BK virus, John Cunningham virus, and human papillomavirus (Widman et al., 2018). However, the antiviral mechanism of SINEs remains to be further studied. A previous study showed that LMB inhibits HCMV replication by blocking the nucleocytoplasmic trafficking of HCMV structural proteins (pp65 and UL94) (Sanchez et al., 2007; Liu et al., 2012). In this study, we examined the effects of Eltanexor (KPT-8602), a newly developed selective inhibitors of nuclear export which showed improved efficacy and *in vivo* tolerability in clinical trials of hematological malignancies (Hing et al., 2016), on HCMV replication. Our results indicate that Eltanexor significantly inhibits HCMV transcript and protein levels during viral lytic infection in fibroblasts. Additionally, Eltanexor targets XPO1 for proteasome-mediated degradation and results in enhanced expression of IFN-β These findings reveal a novel antiviral mechanism of Eltanexor.

## Materials and Methods

### Cells and Viruses

Human foreskin fibroblasts (HFFs) (CRL-4001, ATCC, passages: 10–20) were cultured in Dulbecco’s modified Eagle’s medium (DMEM) supplemented with 10% fetal bovine serum, 100 U/ml penicillin, and 100 μg/ml streptomycin in an incubator with 5% CO_2_ at 37°C. The HCMV strain used was rescued from the HCMV bacterial artificial chromosome (BAC) cosmid termed TB40/E*wt-*mCherry (a generous gift from Eain Murphy at SUNY Upstate Medical University), which has a mCherry marker gene inserted between US34A and TSR1 and replicates like its wild type parent virus as previously described (Sinzger et al., 2008; O’Connor and Shenk, 2011). Viral lysates were pelleted through a D-sorbitol cushion (20%, weight/volume), centrifuged at 20,000 rpm (Hitachi) (equal to 41,224 × g) for 1 h, at 20°C. Viral pellets were resuspended and aliquoted in DMEM, and further titrated by a TCID_50_ assay. To better explain the multiplicity of infection (MOI) in infection assays, viral titers generated by TCID50 assays were expressed as PFU based the formula (1 PFU = 0.69 ^∗^ TCID50).

### Chemicals and Antibodies

Chemicals include Eltanexor (HY-100423, MCE, China) with a purity of 99.71%, polyinosine-polycytidylic acid [poly(I:C)] (B5551, APExBIO Technology, TX, United States), and the proteasome inhibitor MG-132 (S1748, Beyotime, Shanghai, China). Primary antibodies used include: mouse anti-pp52 (UL44) antibodies (0897, sc-58117, Santa Cruz, Dallas, TX, United States), mouse anti-IE1 monoclonal antibody (1B12) and mouse anti-IE2 monoclonal antibody (3H9) (a generous gift from Thomas Shenk at Princeton University), mouse anti-pp65 antibodies (1-L-11, sc-52401, Santa Cruz), mouse anti-pp28 antibodies (CA004, Virusys, MD, United States), rabbit anti-IRF-3 antibodies (11904, Cell Signaling Technologies [CST], Danvers, MA, United States), rabbit anti β-actin antibody (TA-09, ZSGS-Bio, Beijing, China), rabbit anti-pp71 serum (prepared by Chempeptide, Shanghai, China), and rabbit anti-XPO1 monoclonal antibody (D6V7N, CST). Secondary antibodies include horseradish peroxidase (HRP)-conjugated goat anti-mouse immunoglobulin antibodies (7076, CST), HRP-conjugated goat anti-rabbit immunoglobulin antibodies (7074, CST), FITC-conjugated goat anti-rabbit immunoglobulin antibody (SA00003, Proteintech, IL, United States), and Cy3-conjugated goat anti-mouse immunoglobulin antibody (SA00001, Proteintech).

### Cell Viability Assay

HFFs (1 × 10^4^ cells/well) were seeded in 96-well plates and then treated with various concentrations of Eltanexor as indicated and incubated at 37°C for 72 h. Eltanexor was dissolved in dimethyl sulfoxide (DMSO), and an equal volume of DMSO served as a vehicle control. Cell viability was determined by a 3-(4, 5, dimethyliazol-2-yl)-5-(3carboxymethoxy-phenyl)-2-(4-sulfophenyl)-2H-tetrazolium salt (MTS)-based colorimetric assay (Promega) as described by manufacturer’s instructions (Meloni et al., 2015). 50% cytotoxic concentration (CC_50_) is determined according to non-linear trajectory analysis using GraphPad Prism Software (San Diego, CA).

### Eltanexor Add-On and Removal Assays

HFFs (1 × 10^5^ cells/well) were seeded in 12-well plates and infected with HCMV at a MOI = 1. For add-on assays, Eltanexor (0.4 μM) was added at 0, 6, 12, 24, 36, 48, and 60 h post infection (hpi). At 72 hpi, drug-containing medium was replaced with normal drug-free medium. At 96 hpi, culture supernatants were collected for viral titration by a TCID_50_ assay on HFFs. For removal assays, HCMV infected cells were pretreated in medium containing Eltanexor (0.4 μM), and Eltanexor was removed by replacing with normal drug-free medium at 6, 12, 24, 36, 48, 60, and 72 hpi. Culture supernatants were collected at 96 hpi for viral titration by a TCID_50_ assay.

### Quantification of Viral Genome, Viral, and Cellular Transcripts

(i) Quantification of viral genomic DNA from HCMV infected cells. HFFs (3 × 10^5^ cells/well, 6 well plate) were infected with HCMV at a MOI = 1, treated with Eltanexor or vehicle DMSO at indicated concentrations for 1.5 h on ice. Cells were washed with phosphate-buffered saline (PBS) buffer (137 mM NaCl, 3 mM KCl, 8 mM Na2HPO4 [pH 7.5]), trypsinized for 3 min, and centrifuged at 4°C for 3 min at 500 × g. Cells were washed twice with cold PBS and subjected to DNA extraction with DNA extraction kit (D4035, OMEGA, GA, United States). Viral relative genomic copy numbers were quantified by qPCR with UL122 primers and a TB green qPCR Kit (RR420A, TAKARA, Dalian, China). For quantification of viral genomic DNA from the nucleus, cytoplasm/nuclear fractions were isolated with a nuclear and cytoplasmic purification kit (HR0241, BJBALB, Beijing, China) based on manufacturer’s instructions. Viral DNA from cytoplasm/nuclear fractions was extracted and quantified as above. (ii) Quantification of transcripts of viral and cellular genes. Total RNA was extracted from infected HFFs at indicated times with Trizol (9109, TAKARA, Dalian, China). Equivalent amounts (0.1 μg) of RNA were reverse transcribed into cDNA with universal pentamers using a reverse transcription kit (RR820A, TAKARA, Dalian, China). Transcripts of viral genes (UL36, UL122, UL123, UL44, UL82, UL83, UL99, and UL75) and cellular genes (IFN-β1, ISG15, ISG54) were further quantified by qPCR. β-actin served as an internal control. All primers used are listed in Table 1.

**TABLE 1:** Primers used in this study.

Primer	Sequence
UL123(F)	5′ GCCTTCCCTAAGACCACCAAT 3′
UL123(R)	5′ ATTTTCTGGGCATAAGCCATAATC 3′
UL44(F)	5′ TACAACAGCGTGTCGTGCTCCG 3′
UL44(R)	5′ GGCGTGAAAAACATGCGTATCAAC 3′
UL99(F)	5′ GTGTCCCATTCCCGACTCG 3′
UL99(R)	5′ TTCACAACGTCCACCCACC 3′
UL122(F)	5′ TGTTCCGTCACACCAATCGTTCTC 3′
UL122(R)	5′ AGGCGACACCGTACCTGATCC 3′
UL36(F)	5′ GGCACCGTCTGTTCGCAAGG 3′
UL36(R)	5′ CCGATGAGCACGATGAGTTGGTAG 3′
UL75-F	5′ TCTTGACGCCGCACTTGACTTC 3′
UL75-R	5′ CATCTGACATCGACCGCTCTTGAG 3′
UL82-F	5′ GCGAGCCTTGACGACTTGGTAC 3′
UL82-R	5′ GAAGTGGAAGCGGTGCTGATGG 3′
Actin(F)	5′ TCCTCCTGAGCGCAAGTACTC 3′
Actin(R)	5′ CGGACTCGTCATACTCCTGCTT 3′
UL83(F)	5′ AGGTGCAGCACACGTACTTT 3′
UL83(R)	5′ TAGTGGTGCACGTTGATGCT 3′
IFNβ1(F)	5′AAACTCATGAGCAGTCTGCA 3′
IFNβ1(R)	5′AGGAGATCTTCAGTTTCGGAGG 3′
ISG15(F)	5′ ATGGGCTGGGACCTGACG 3′
ISG15(R)	5′ GCCAATCTTCTGGGTGATCTG 3′
ISG54(F)	5′ AACCTACTGGCCTATCTAAAGC 3′
ISG54(R)	5′ CATGCTCTTGCTGGATTAACTC 3′

### Western Blotting Assay

Cells were lysed in RIPA buffer (50mM Tris (pH 7.4), 150mM NaCl, 1% Triton X-100, 1% sodium deoxycholate, 0.1% SDS) (BL504A, Biosharp, China) supplemented with proteinase inhibitors (4693159001, Roche, Basel, Switzerland). Equal amounts of proteins were separated by sodium dodecyl sulfate-polyacrylamide gel electrophoresis (SDS-PAGE), transferred and immobilized on polyvinylidene fluoride (PVDF) membranes. The membrane was blocked with 5% skim milk in PBST (PBS plus 0.05% Tween 20) for 1 h, followed by overnight incubation with rabbit or mouse primary antibodies for the proteins of interest. Blots were washed three times with PBST for 15 min each, followed by incubation with HRP-conjugated secondary antibodies for 2 h. Blots were then washed with PBST three times and developed with the ECL enhanced chemiluminescence system (G3308, Gbcbio Technologies Inc., Guangzhou, China). Images were taken and analyzed by Amersham Imager 600 system (GE healthcare, United States).

### Indirect Immunofluorescence Assay

HFFs (1 × 10^5^ cell/well, 12 well plate) were seeded on coverslips and infected with HCMV at a MOI = 1, fixed at indicated times with 4% paraformaldehyde for 15 min at room temperature, and then permeabilized with 0.1% Triton X-100 in PBS for 15 mins. The coverslips were then washed three times with PBST before being blocked with PBST containing 5% bovine serum albumin for 30 min. The cells were then incubated with primary antibodies (mouse anti-pp65, rabbit anti-IRF3) for 1 h. After washing three times with PBST, cells were incubated for another hour with FITC or Cy3-conjugated secondary antibodies. Slides were washed three times with PBST and mounted with anti-fade reagent with DAPI (4’, 6-diamidino-2-phenylindole dihydrochloride) (28718-90-3, Biofroxx, United Kingdom). Images were taken with a DeltaVision Elite microscope (GE healthcare, United States).

### Enzyme-Linked Immunosorbent Assay (ELISA)

HFFs (1 × 10^5^ cells/well) were seeded in 12-well plates and infected with HCMV at MOI = 1 or mock infected on ice for 1.5 h. Cells were then treated with Eltanexor (0.4 μM) or DMSO. Similarly, HFFs (1 × 10^5^ cells/well) were treated with Poly (I:C) (20μg/mL) with or without Eltanexor (0.4 μM), and then culture supernatants were collected at indicated time points. Production of IFN-β in supernatants was determined with an ELISA kit (KE1364, ImmunoWay, TX, United States).

### Statistical Analysis

Statistical significance was assessed with GraphPad Prism 5.0 software. Student’s *t*-tests were used to determine differences between the means of two groups. Multiple-comparison significance was determined by two-way ANOVAs. In all analyses, two-sided *p*-values were used, and *p* < 0.05 was considered statistically significant, ^∗^*p* < 0.05, ^∗∗^*p* < 0.01, ^∗∗∗^*p* < 0.001.

## Results

### Eltanexor Inhibits HCMV Lytic Replication

**FIGURE 1:**
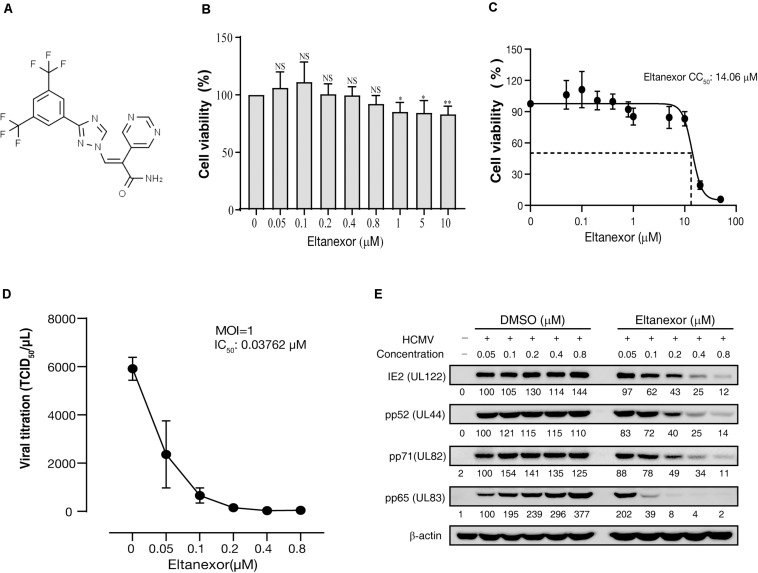
Eltanexor inhibits HCMV lytic replication in HFFs in a dose-dependent manner. **(A)** Structure of Eltanexor (KPT-8602). **(B)** Effects of Eltanexor on cell viability were assayed at 72 h post treatment (hpt). HFFs were treated with Eltanexor at indicated concentrations or vehicle (DMSO, 0 μM). Cell viability was analyzed by a MTS-based colorimetric assays at 72 hpt. Data is presented as% of cell viability relative to vehicle control (indicated by 0 μM). Values represent mean ± SEM; *n* = 5. Statistical analyses were performed between indicated concentrations and 0 μM. ^∗^*p* < 0.05, ^∗∗^*p* < 0.01. **(C)** 50% cytotoxic concentration (CC50) is determined at 14.06 mM based on the results of cell viability assays according to non-linear trajectory analysis using GraphPad Prism. **(D)** Eltanexor inhibits the production of infectious virions in a dose-dependent manner. HFFs were infected with HCMV at a MOI = 1 and treated with the indicated concentrations of Eltanexor. Culture supernatants were collected at 96 hpi and further titrated by a TCID_50_ method. Values represent mean ± SEM; *n* = 3. **(E)** Eltanexor inhibits viral protein synthesis in a dose-dependent manner. Cells were infected with HCMV at a MOI = 1 and then treated with DMSO or Eltanexor at the indicated concentrations for 72 h. Cell lysates were collected and viral protein expression was assayed by immunoblot for the following proteins: IE2, pp52, pp71, and pp65. Intensity values of viral protein bands were analyzed by Amersham Imager 600 analysis software. The experiments were repeated three times.

Eltanexor (KPT-8602) is a newly developed synthetic second-generation XPO1 inhibitor (Figure 1A) and less toxic than analogs, and is currently in phase I/II clinical trials for multiple myeloma (Cornell et al., 2016). Therefore, we aim to examine its effects on HCMV replication. Firstly, we analyzed the toxicity of Eltanexor on HFFs. Eltanexor does not significantly affect cell viability at concentrations less than or equal to 0.8 μM (Figure 1B), and 50% cytotoxic concentration (CC_50_) is determined at 14.06 μM (Figure 1C). Therefore, we examined the effect of Eltanexor on HCMV replication at concentrations between 0 and 0.8 μM as indicated. Eltanexor inhibits the production of HCMV progeny virions in a dose-dependent manner, and the half-maximal inhibitory concentration (IC_50_ or EC_50_) is determined at 0.03762 μM (Figure 1D). Selectivity of Index (SI) of Eltanxor is calculated as 374. Western-blotting assays also show Eltanexor treatment inhibits the expression of IE2/86 (encoded by UL122), early protein pp52 (UL44), and late proteins pp71 and pp65 (UL82 and UL83) in a dose-dependent manner (Figure 1E). Taken together, these results demonstrate that Eltanexor inhibits HCMV replication in a dose-dependent manner.

### Eltanexor Does Not Affect Viral Entry or Nuclear Import of Genomic DNA

**FIGURE 2:**
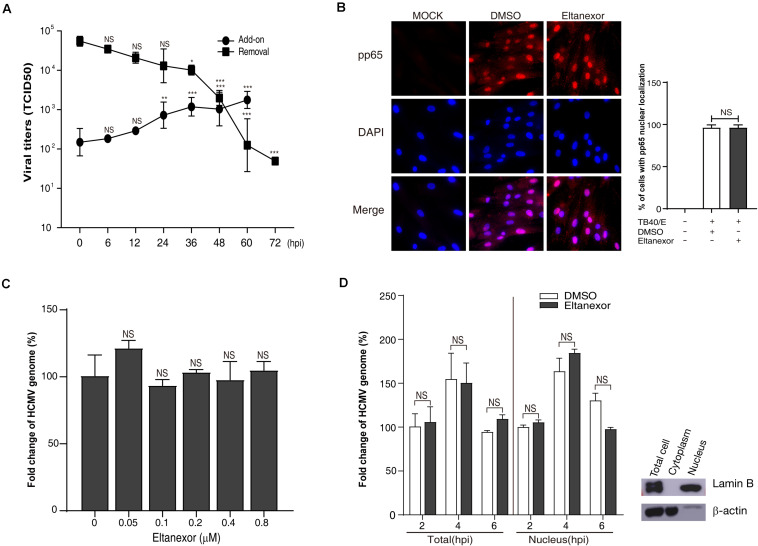
Eltanexor treatment does not affect viral entry or nuclear trafficking. **(A)** Eltanexor add-on and removal assays showed viral replications was suppressed after 24 hpi. Culture supernatants collected from add-on or removal assays and HCMV titers were determined by a TCID_50_ assay. Data represent mean ± SEM, *n* = 3. Statistical analyses were performed between indicated hpi and 0 hpi, *p < 0.05, **p < 0.01, ***p < 0.001. **(B)** Nuclear localization of viral tegument protein pp65 is not affected by Eltanexor treatment. HFFs were infected with HCMV at a MOI = 1 and treated with Eltanexor (0.4 μM) or DMSO. At 6 hpi, cells were fixed and immunostained with mouse antibodies against pp65, followed by Cy3-conjugated goat anti-mouse antibody (red). Nuclei were stained with DAPI (blue). Percentages of cells with pp65 nuclear localization were calculated based on counts from 5 random fields with at least three hundred cells. The experiments were repeated 3 times. Data represent mean ± SEM, *n* = 3. NS stands for not significant. **(C)** Entry of the viral genome is not affected by Eltanexor treatment. HFFs were infected with HCMV at a MOI = 1 and treated with Eltanexor at different concentrations or DMSO for 2 h. Viral genomic DNA was isolated and quantified by qPCR with primers for UL122. Fold changes of viral genome in HCMV infected cells treated with 0 μM of Eltanexor were normalized to 100%. Statistical analyses were performed between different concentrations of Eltanexor and vehicle (0 mM). Data represent mean ± SEM, *n* = 3. **(D)** Nuclear trafficking of viral genomic DNA is not affected by Eltanexor treatment. HFFs were infected with HCMV at a MOI = 1 and then treated with Eltanexor (0.4 μM) or DMSO. Viral DNA isolated from total cells or nuclei at 2, 4, and 6 hpi were quantified by qPCR with primers for UL122. Fold changes of viral genome in HCMV infected cells treated with DMSO at 2 hpi were normalized to 100%. Data represent mean ± SEM, *n* = 3. Statistical analyses were performed between Eltanexor and DMSO treatments. Total cell lysates, cytoplasm and nuclear components were isolated with commercial cytoplasm-nuclear isolation kits and purity was confirmed by immunoblot against the nuclear marker Lamin B and the cytoplasmic marker β-actin. The experiments were repeated three times.

HCMV replication is a complex process consisting of multiple steps, including attachment and entry, intracellular trafficking, uncoating, genomic replication, protein synthesis, capsid assembly and DNA encapsidation, translocation, envelopment, and egress (Edward et al., 2013). To determine at which step of HCMV replication was interrupted by Eltanexor treatment, Eltanexor add-on and removal assays were performed. The results of add-on assays indicate the production of progeny virions significantly escapes the suppression of Eltanexor treatment when no drug is added before 24 hpi; on the other hand, the results of removal assays indicate the production of progeny virions is inhibited when Eltanexol is removed from Eltanexor pretreated medium later than 36 hpi (Figure 2A). To further examine whether Eltanexor affects viral entry at early stages of HCMV infection, localization of viral tegument protein pp65 delivered as part of the virions was determined by immunofluorescence assay. The nuclear translocation of pp65 is not significantly affected by Eltanexor treatment (Figure 2B). Quantification of viral genomic DNA in Eltanexor treated HFFs shows viral attachment is not significantly affected in a dose-dependent manner (Figure 2C). Furthermore, quantifications of viral genomic DNA isolated from nuclear fractions and wholes cell lysates show that Eltanexor does not affect nuclear import of viral genomic DNA (Figure 2D). From these results, we conclude that Eltanexor does not significantly affect viral entry and nuclear import of viral genomic DNA but can exert inhibitory effects within 24–36 hpi.

### Eltanexor Treatment Decrease the Transcript and Protein Levels of Viral Immediate-Early (IE2), Early (E), and Late (L) Genes

**FIGURE 3:**
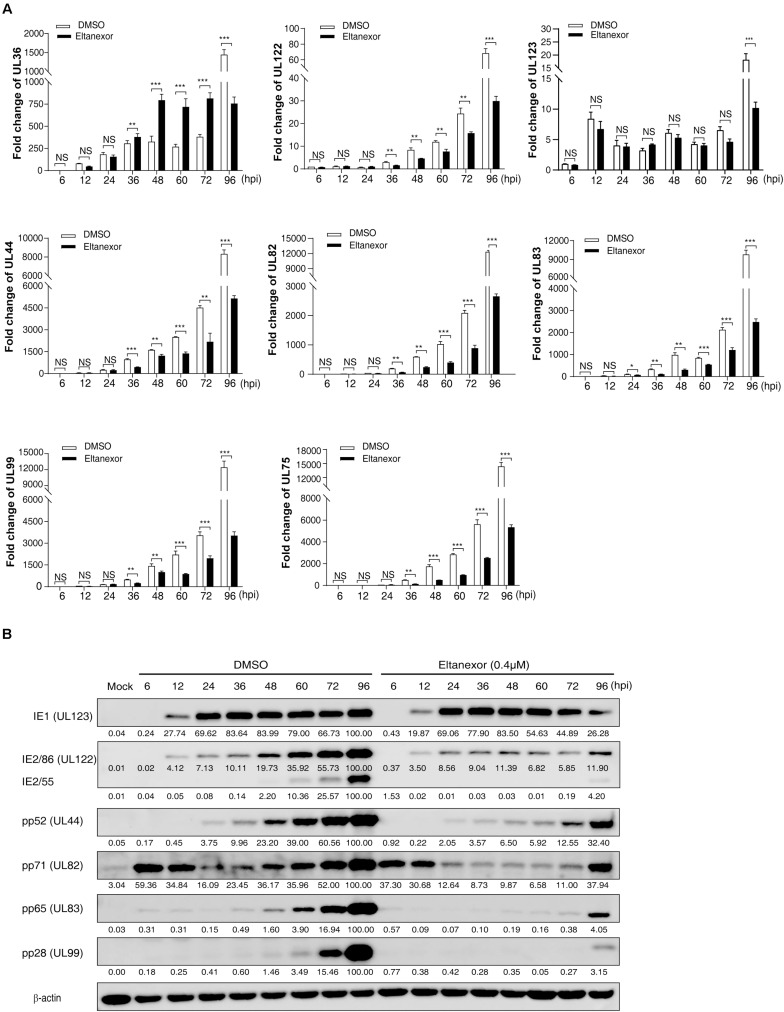
Transcript and protein levels of HCMV are suppressed by Eltanexor treatment in HFFs. **(A)** Transcript levels of HCMV IE genes (UL36, UL122, and UL123), E gene (UL44), and L genes (UL82, UL83, UL99 and UL75) were quantified by RT-qPCR at the indicated time points. HFFs were infected with HCMV at a MOI = 1 and then treated with Eltanexor (0.4 μM) or DMSO. The levels of viral transcripts at the indicated time points were determined by RT-qPCR. Fold changes of viral transcripts were normalized to the internal control β-actin. Fold changes of viral transcripts in HCMV infected cells at 6 hpi were further normalized to 1 (arbitrary unit). Data represent mean ± SEM, *n* = 3, *p < 0.05, **p < 0.01, ***p < 0.001. **(B)** protein levels of IE, E, and L genes were measured by Western-blotting at the indicated time points. Cell lysates were collected at the indicated time points as described in (A) and immunoblotted with antibodies against viral IE proteins (IE1 and IE2, encoded by UL123 and UL122), viral E protein (pp52, encoded by UL44), and viral L proteins (pp71, pp65, and pp28, encoded by UL82, UL83, and UL99). β-action served as the loading control. Viral protein band intensities were analyzed by Amersham Imager 600 analysis software. The experiments were repeated three times.

Since Eltanexor does not affect HCMV entry and trafficking, we speculate that it might affect viral transcript and protein levels. Hence, we examined the transcript levels of viral genes under Eltanexor treatment (0.4 μM). The transcript levels of IE genes (UL36, UL122, and UL123), and E (UL44) and L genes (UL82, UL83, UL99, and UL75) (Landolfo et al., 2003) were quantified by RT-qPCR at 6, 12, 24, 36, 48, 60, 72, and 96 hpi. Eltanexor treatment show distinct effects on the transcript levels of three IE genes (UL36, UL122, and UL123). Eltanexor treatment increases the transcript levels of UL36 after 36 hpi, and appears not affect the transcript levels of UL123 throughout the first replication cycle (within 72 hpi), but reduce the transcript levels of UL122 after 36 hpi. Additionally, the transcript levels of E (UL44) and L (UL82, UL83, UL99, and UL75) genes are inhibited after 36 hpi (Figure 3A), compared with DMSO controls. To further determine whether Eltanexor treatment suppresses viral protein expression, Western-blotting assays were performed. The IE1 does not appear to be suppressed, but IE2 is significantly reduced by Eltanexor in comparison to DMSO controls. The protein levels of E protein pp52 (encoded by UL44), pp65 (UL83), pp71 (UL82), and L protein pp28 (UL99) are suppressed after 36 hpi (Figure 3B). These results are consistent with the transcript levels of these genes observed in Figure 3A. In summary, our results indicate that Eltanexor treatment suppresses the protein levels of IE2 as well as other early and late viral proteins. The major IE protein IE2, encoded by UL122, is a crucial activator of viral late genes (Marchini et al., 2001). IE1, encoded by UL123, primarily functions as a coactivator with IE2 (Ahn and Hayward, 2000). Both IE1 and IE2 are derived from a same transcript but from two different spliced isoforms (Stinski and Petrik, 2008). the transcription of IE1 is less affected by Eltanexor treatment than IE2, likely because it is processed prior to IE2. Whether IE2 splice processing is inhibited by Eltanexor at the early stage of infection remains to be further determined.

### Eltanexor Treatment Targets XPO1 for Proteasome-Mediated Degradation in HCMV Infected HFFs

**FIGURE 4:**
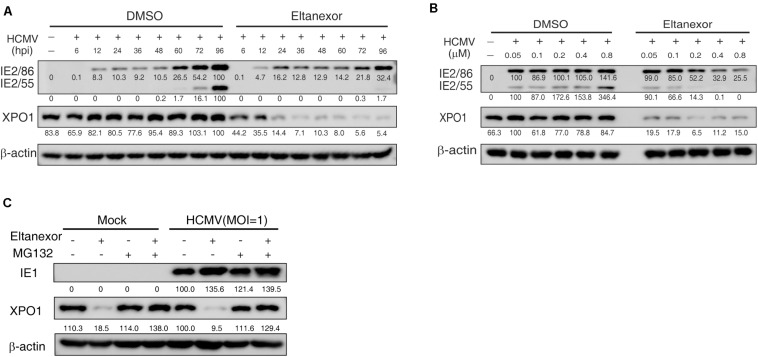
Eltanexor treatment suppresses viral protein synthesis by targeting XPO1 for proteasome-mediated degradation. **(A)** Eltanexor treatment decreases XPO1 protein levels during the course of HCMV infection. HFFs were infected with HCMV at a MOI = 1 and treated with Eltanexor (0.4 μM) or DMSO. Cell lysates were collected at the indicated time points (hpi) and assayed by immunoblot for IE2, XPO1, and β-action. Protein band intensities were analyzed by Amersham Imager 600 analysis software. **(B)** Eltanexor treatment decreases the protein levels of XPO1 in a dose-independent manner. HFFs were infected with HCMV at MOI = 1 and treated with Eltanexor at the indicated concentrations or equal volumes of vehicle (DMSO). Cell lysate were collected at 72 hpi and immunoblotted and analyzed as in **(A)**. **(C)** Eltanexor induced XPO1 degradation is inhibited by the proteasome inhibitor MG-132. HFFs were infected with HCMV at a MOI = 1 on ice for 1.5 h, and then grown in complete medium with Eltanexor (0.4 μM) and/or the proteasome inhibitor MG132 (5μM). Cell lysates were collected and assayed by immunoblot for IE1, XPO1, and β-actin.

To investigate the mechanism of how Eltanexor treatment suppresses HCMV replication, we examined whether Eltanexor directly targets XPO1. The protein levels of XPO1 appears not apparently upregulated in HCMV infection at indicated time points (Figure 4A), unlike in leukemia and other cancers (Vercruysse et al., 2017; Azizian and Li, 2020). During the course of HCMV infection (0∼96 hpi), the degradation of XPO1 was observed after 6 h post treatment with Eltanexor, but not in vehicle (DMSO)-treated control cells (Figure 4A). Eltanexor treatment decreases XPO1 levels, which is not dose-dependent (Figure 4B). When Eltanexor-treated cells is additionally treated with the proteasome inhibitor MG132 (5μM), the degradation of XPO1 is completely inhibited (Figure 4C). These results suggest that Eltanexor treatment inhibits HCMV replication by targeting XPO1 for degradation in a proteasome-dependent manner.

### Eltanexor Treatment Increases the Nuclear Accumulation of Interferon Regulatory Factor 3 (IRF-3)

**FIGURE 5:**
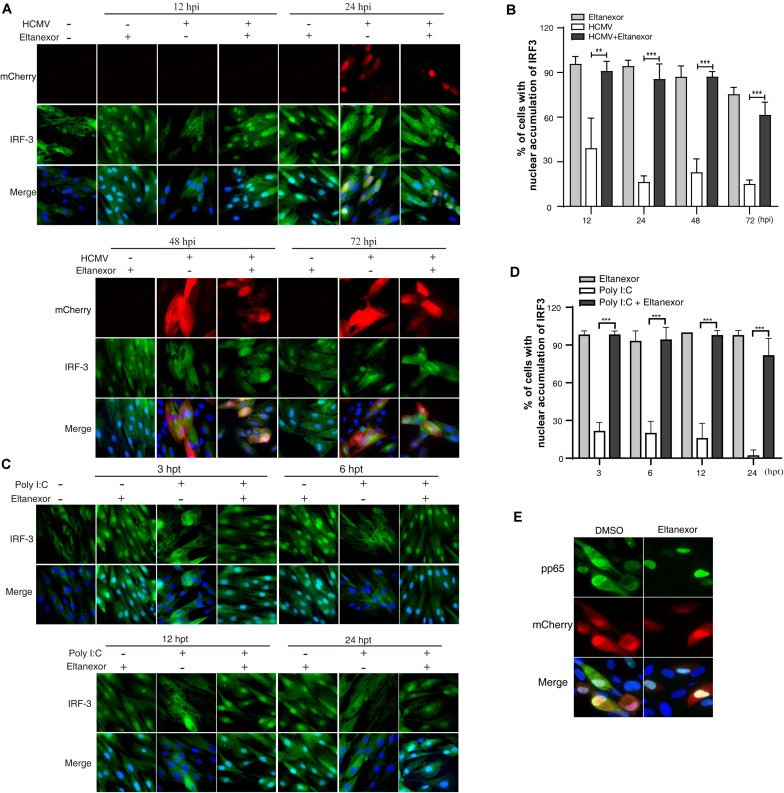
Eltanexor treatment increases nuclear retention of IRF3 in HCMV infected or Poly (I:C) treated HFFs. **(A)** HFFs were infected with HCMV at a MOI = 1 and treated with Eltanexor (0.4 μM) or vehicle DMSO. At different time points, HFFs were fixed and immunostained with rabbit anti-IRF-3 antibody followed by FITC-conjugated goat anti-rabbit antibody (green). Nuclei were stained with DAPI (Blue). HCMV infected cells were indicated by mCherry (red). **(B)** Percentages of cells showing nuclear accumulation of IRF-3 in DMSO or Eltanexor treated HCMV-infected cells in **(A)** were calculated based on 5 random fields with more than 300 cells. The experiments were repeated 3 times. Data represent mean ± SEM, *n* = 3, **p < 0.01, ***p < 0.001. **(C)** Eltanexor treatment increases the nuclear accumulation of IRF-3 in poly (I:C) treated HFFs. Poly (I:C) was added the culture medium at a concentration of 20μg/mL. HFFs were fixed and immunostained for IRF-3 as described in **(A)**. **(D)** Percentages of cells showing nuclear accumulation of IRF-3 in **(C)** were calculated based on 5 random fields. Data represent mean ± SEM; *n* = 5, ***p < 0.001. **(E)** Nuclear retention of viral tegument protein pp65 in Eltanexor treated HFFs at 72 hpi. HFFs were infected with HCMV at a MOI = 1, and treated with Eltanexor (0.4 μM) or vehicle DMSO. Cells were immunostained for pp65 (green). Nuclei were stained with DAPI (blue). HCMV infected cells were indicated by mCherry (red). The experiments were repeated 3 times.

Upon HCMV infection, cytoplasmic IRF-3 is phosphorylated and dimerized, and then translocated from the cytoplasm into the nucleus (DeFilippis et al., 2006). The localization of IRF-3 is mediated by both a nuclear localization sequence (NLS) and a nuclear export sequence (NES) that are recognized by distinct shuttling receptors. The NLS and NES in IRF-3 are both constitutively active, but nuclear export is normally dominant. IRF-3 is exported from the nucleus to the cytoplasm via XPO1 (Kumar et al., 2000). Since Eltanexor treatment induces XPO1 degradation (Figures 4A–C), we speculate that it might increase nuclear localization of IRF-3 in HCMV infected cells. To investigate this hypothesis, we examined the localization of IRF-3 by immunofluorescence assays in HCMV infected HFFs treated with Eltanexor or DMSO (Figure 5A). When examined at 12, 24, 48, and 72 hpi, Eltanexor treatment increases the percentage of HCMV infected cells with nuclear localization of IRF-3 (Figure 5B). HCMV infection causes about 30% of cells to exhibit IRF3 nuclear localization, while Eltanexor treatment alone leads to nearly 100% cells with nuclear localization of IRF3. Viral dsRNA is a potent toll-like receptor 3 (TLR3) agonist that stimulates nuclear translocation of phosphorylated IRF3 and induces a type I interferon (IFN) response (Kumar et al., 2006; Murshid et al., 2016). Eltanexor treatment (0.4 μM) also increased the nuclear localization of IRF-3 in cells treated with a substitute dsRNA, polyinosine-polycytidylic acid [poly(I:C)] (Figures 5C,D), similar to that in HCMV-infected cells. In addition, Eltanexor treatment also inhibits the nuclear export of viral tegument protein pp65 which also contains a NES (Figure 5E), which was in line with a previously published study (Sanchez et al., 2007). Our results suggest that Eltanexor treatment increases nuclear retention of cellular IRF-3.

### Eltanexor Treatment Increases Type I Interferon (IFN-I) Production in HCMV Infected HFFs

**FIGURE 6:**
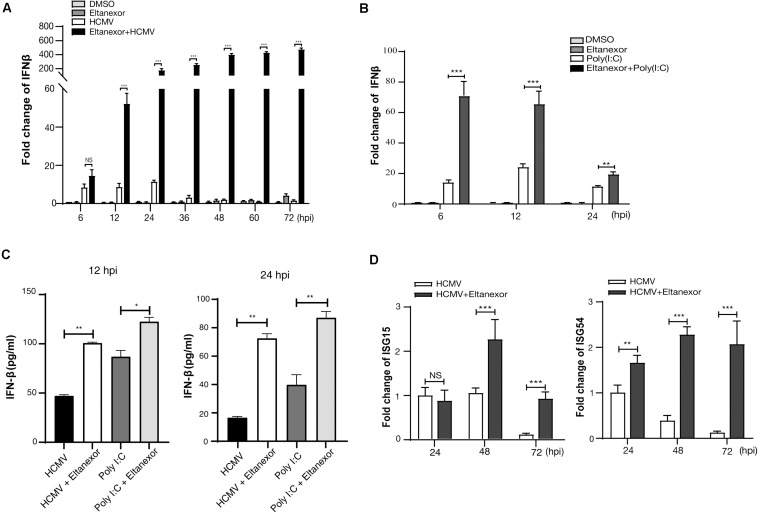
Eltanexor treatment enhances a type I IFN response in HCMV-infected or poly (I:C) treated HFFs. **(A)** Transcriptional levels of IFN-β in HCMV infected cells treated with Eltanexor or DMSO were determined by RT-qPCR. HFFs were infected with HCMV at a MOI = 1, and treated with Eltanexor (0.4 μM) or DMSO. IFN-β mRNA levels were quantified by RT-qPCR at the indicated timepoints and normalized to cellular β-actin. Fold changes of IFN-β in DMSO-treated samples at 6 hpi were normalized to 1 (arbitrary unit). Data represent mean ± SEM, *n* = 3. **(B)** Eltanexor increase the transcriptional levels of IFN-β in poly(I:C) treated cells. HFFs were treated with DMSO, Eltanexor (0.4 μM), poly(I:C) (20μg/mL), or Eltanexor and poly(I:C). mRNA levels of IFN-β were quantified by RT-qPCR at the indicated timepoints and normalized to cellular β-actin. Data represent mean ± SEM, *n* = 3. **(C)** The production of IFN-β in the culture supernatants from HCMV infected or Poly (I:C) treated HFFs was determined by ELISA at the indicated timepoints. Data represent mean ± SEM, *n* = 3. **(D)** Expression of ISG15 and ISG54 in HCMV infected HFFs with or without Eltanexor treatment. Cells were infected with HCMV (MOI = 1) and treated with Eltanexor (0.4 μM) or vehicle DMSO. Transcript levels of ISG15 and ISG54 were determined by RT-qPCR at the indicated timepoints. Fold changes of transcripts in HCMV infected cells at 24 hpi were normalized to 1 (arbitrary unit). Data represent mean ± SEM, *n* = 3. *p < 0.05, **p < 0.01, ***p < 0.001.

We next examined whether the nuclear accumulation of IRF3 induced by Eltanexor treatment could translate into increased production of type I IFN. Our results indicate that Eltanexor treatment (0.4 μM) increases the transcript levels of IFN-β in HCMV infected HFFs but not in uninfected cells at the indicated time points (Figure 6A). Similarly, Eltanexor treatment increases transcript levels of IFN-β in poly(I:C) treated cells but not in untreated cells (Figure 6B). However, Eltanexor alone in mock infected cells does not increase transcript levels of IFN-β, which is not consistent with the nuclear accumulation of IRF3 as observed in Figure 5. The protein levels of IFN-β in culture supernatants of HCMV infected or poly (I:C) treated cells determined by ELISA (Figure 6C) are in line with transcript levels of IFN-β (Figures 6A,B). Furthermore, transcript levels of interferon-stimulated genes ISG15 and ISG54 are also increased by Eltanexor treatment in HCMV infected cells at indicated time points (Figure 6D). Eltanexor alone could not stimulate IFN-β production, despite the nuclear accumulation of IRF3 as observed in Figure 5, which probably is due to lack of other transcriptional activators to form transcription initiation complexes, and thus is not sufficient to initiate the transcription of IFN-β. These results indicate Eltanexor increases the type I interferon response to inhibit HCMV replication.

## Discussion

The infection cycle of many viruses relies on XPO1 for transporting viral and cellular components from the nucleus to the cytoplasm (Mathew and Ghildyal, 2017). Interruption of nuclear export by selective inhibitors of nuclear export (SINEs) results in the reduction of viral protein expression and viral DNA replication, incomplete viral assembly, and proinflammatory responses (Elton et al., 2001; Pasdeloup et al., 2005; Cao and Liu, 2007; Sanchez et al., 2007; Ghildyal et al., 2009; Nakano and Watanabe, 2016). Eltanexor, as a second-generation inhibitor of XPO1, has been demonstrated to substantially reduce brain penetration of hematopoietic malignancies with minimal toxicity to normal hematopoietic stem and progenitor cells compared to other SINEs (Das et al., 2015). Our study shows Eltanexor inhibits HCMV replication with better efficiency (EC_50_ = 0.03762 μM, CC_50_ = 14.06 μM, SI = 374) than Verdinexor (EC50 = 2.5 μM, CC_50_ = 73.3 μM, SI = 29) (Widman et al., 2018). In the preclinical study of Eltanexor (KPT8602) treating acute myeloid leukemia (Hing et al., 2016), the mean plasma concentrations reached 3 μM in primates orally dosed at 10 mg/kg, which is way higher than EC50 (0.03762 μM) for HCMV, suggesting Eltanexor might be suitable for anti-HCMV clinical trial in primates. The limitation of this study is that only single recombinant HCMV strain was used for examining EC50, CC50, and SI. The index for resistant HCMV strains and other viruses can vary and further tests are warranted. Mechanistically, we have found that Eltanexor reduces the transcript and protein levels of IE2 (UL122) and other early and late genes post entry at the early stage of infection (within 24–36 hpi). In addition, Eltanexor treatment induces proteasome-mediated degradation of XPO-1 (Figure 4), increases the nuclear retention of IRF3 (Figure 5), and ultimately promotes production of IFN-β (Figure 6), which represents a novel antiviral mechanism of Eltanexor.

XPO1 overexpression is a common feature among many human cancer types, including lymphoma pancreatic, ovarian, glioma, lung, gastric, prostate, and colorectal cancers, and is associated with poor prognosis (Azizian and Li, 2020). Although suppression of XPO1-mediated nuclear export presents a unique therapeutic strategy, side effects of XPO1 inhibitors remain unclear. On the other hand, viral infection does not affect the expression levels of XPO1 as observed in this study (Figures 4A,B). The antiviral mechanism of SINEs is not fully understood, presumably by interrupting nuclear export of XPO1’s cargo proteins at various stages of the viral lifecycle (Mathew and Ghildyal, 2017). Eltanexor directly inhibits XPO1-mediated nuclear export of viral proteins carrying a NES. HCMV replication is highly dependent on XPO1 for exporting both viral and cellular proteins of the nucleus (Sanchez et al., 2007; Frankenberg et al., 2012; Liu et al., 2012). Previous studies show the prototype XPO-1 inhibitor LMB impairs nuclear shuttling of viral proteins pp65 (Sanchez et al., 2007; Frankenberg et al., 2012). At the late phase of the lytic infection cycle, pp65 utilizes XPO1-mediated transport to shuttle from the nucleus to the cytoplasm, and disruption of XPO1-medited export of pp65 by LMB results in its nuclear retention, and dampens viral replication (Sanchez et al., 2007). Tegument protein UL94 is another late protein which relies on XPO1 mediated nuclear export (Liu et al., 2012). Other tegument proteins such as pp71, UL35, UL47, and UL48, structural proteins pp150, pp28, and pIRS1/pTRS1, and non-structural protein UL97 are also translocated from the nucleus to the cytoplasm and play crucial roles in transcription of the viral genome, translocation of maturing nucleocapsids from the nucleus to the cytoplasm, modifying host cell response to infection, interfering with host cell cycle and optimizing the nuclear environment (Kalejta, 2008). XPO1 mediated nuclear export of viral proteins mentioned above normally occurs at the late stages of viral infection cycle (after 48 hpi). However, our results from Eltanexor add-on and removal assays show that Eltanextor treatment can significantly inhibit HCMV replication at the early stages of infection (within 24–36 hpi) (Figure 2). In particular, the expression of IE2 (UL122) is inhibited by Eltanexor treatment (Figure 3) at early times of infection, which can impede the progression of the rest of viral replication lifecycle. Thus, we speculate that Eltanexor exerts antiviral effects through targeting cellular or viral proteins that might impede the splicing or transcription of IE2 at the immediate-early and early stages of HCMV replication.

A recent deep proteomic study identified more than 1,000 proteins exported by XPO1 (Kirli et al., 2015), which means antiviral mechanisms of Eltanexor are complicated and might impact many signaling pathways. Eltanexor was reported to block cell proliferation and growth transformation by inducing p53-mediated cell cycle arrest in Kaposi’s sarcoma-associated herpesvirus-transformed cells (Gruffaz et al., 2019). In addition, Yang et al. (2014) reported XPO1 inhibitors induced nuclear accumulation of TP53, leading to apoptosis of human melanoma cells. Hence, it is reasonable to postulate that Eltanexor treatment induce nuclear accumulation of p53 and enhance apoptosis in HCMV infected cells. Interestingly, upregulation of immediate early viral protein UL36 (Figure 3A) which encodes a viral inhibitor of caspase 8 induced apoptosis, might be a feedback response to counteract the pro-apoptotic effects of Eltanexor.

In this study, we found that Eltanexor treatment directly induced proteasome mediated degradation of XPO1 (Figures 4A–C) in HCMV infected cells. These results were consistent with XPO1 degradation in Eltanexor treated chronic lymphocytic leukemia cells (Hing et al., 2016). While many cellular proteins utilize XPO1, of particular interest to HCMV infection is IRF3, which relies solely on XPO1 for its nuclear export (Kumar et al., 2000). Type I IFNs are the first line of innate immune defense against HCMV infection. HCMV infection recruits a transcriptional complex containing interferon regulatory factor 3 (IRF-3) and acetyl transferases [CREB-binding protein (CBP) and p300] to the nucleus and thus stimulates type I IFN production (DeFilippis et al., 2006). Our results indicate Eltanexor treatment leads to increased nuclear accumulation of IRF3 in HCMV infected cells (Figures 5A,B), which contributes to increased expression of IFNβ as well as ISG15 and ISG54 (Figure 6). Therefore, we hypothesize that increased IFN-I production represents a novel antiviral mechanism of Eltanexor at the early stage of infection. A recent study showed XPO1 inhibition induces retention of autophagy adaptor protein p62 (SQSTM1) in the nucleus, which enhances activation of TBK1 and IRF3, resulting in increased expression of innate immune-related genes including IRF7, ISG15, IFIT1, IFIT2, and IFIT3, leading to a reduction of KSHV lytic replication (Meng and Gao, 2021). Thus, we speculate Eltanexor has potential for blocking a broad spectrum of viral pathogens through promoting type I interferon response.

XPO1 is considered a reasonable target for cancer therapy because overexpressed XPO-1 in cancers causes nuclear mislocalization of tumor suppressors and other regulatory proteins (Wang and Liu, 2019). The mechanisms of how Eltanexor targets cancer and viral pathogens might be quite different, and cautions should be taken for the side effects of Eltanexor in animal experiments and clinical trials of anti-cancer and antiviral therapies.

## Data Availability Statement

The raw data supporting the conclusions of this article will be made available by the authors, without undue reservation.

## Author Contributions

QQ: conceptualization, writing—review and editing, supervision, project administration, and funding acquisition. YL: investigation, data curation, and writing—original draft preparation. YL, XK, TD, and QQ: formal analysis. YL, XK, and TD: validation. YL and QQ: methodology. YL and XK: software. YL and TD: visualization. All authors contributed to the article and approved the submitted version.

## Conflict of Interest

The authors declare that the research was conducted in the absence of any commercial or financial relationships that could be construed as a potential conflict of interest.
